# Supplementary material for: What is the impact of multimorbidity on the risk of hospitalisation in older adults? A systematic review study protocol
Source: BMJ Open. 2021 Jun 22;11(6):e049974. doi: 10.1136/bmjopen-2021-049974 (PMC8230920; doi:10.1136/bmjopen-2021-049974)
Supplement: Author's manuscript [file bmjopen-2021-049974.draft_revisions.pdf]

BMJ Open

BMJ Open is committed to open peer review. As part of this commitment we make the peer review history of every article we publish publicly available.

When an article is published we post the peer reviewers' comments and the authors' responses online. We also post the versions of the paper that were used during peer review. These are the versions that the peer review comments apply to.

The versions of the paper that follow are the versions that were submitted during the peer review process. They are not the versions of record or the final published versions. They should not be cited or distributed as the published version of this manuscript.

BMJ Open is an open access journal and the full, final, typeset and author-corrected version of record of the manuscript is available on our site with no access controls, subscription charges or pay-per-view fees (http://bmjopen.bmj.com).

If you have any questions on BMJ Open's open peer review process please email info.bmjopen@bmj.com

BMJ Open

What is the impact of multimorbidity on the risk of hospitalization in older adults? A systematic review study protocol

Journal:	*BMJ Open*
Manuscript ID	bmjopen-2021-049974
Article Type:	Protocol
Date Submitted by the Author:	06-Feb-2021

Complete List of Authors:	Rodrigues, Luciana; Universidade Federal de Goiás, Graduate Program in Health Sciences<br>Rezende, Andréa; Universidade Federal de Goias, Graduate Program in Health Sciences<br>Nogueira, Letícia; Universidade Federal de Goiás, Graduate Program in Health Sciences<br>Nunes, BP; Universidade Federal de Pelotas, Department of Nursing<br>de Oliveira, Cesar; University College London, Epidemiology and Public Health<br>Noll, Matias; Instituto Federal Goiano<br>Silveira, Erika; Universidade Federal de Goias, Faculdade de Medicina

Keywords:	GERIATRIC MEDICINE, EPIDEMIOLOGY, PUBLIC HEALTH

SCHOLARONE™
Manuscripts

I, the Submitting Author has the right to grant and does grant on behalf of all authors of the Work (as defined in the below author licence), an exclusive licence and/or a non-exclusive licence for contributions from authors who are: i) UK Crown employees; ii) where BMJ has agreed a CC-BY licence shall apply, and/or iii) in accordance with the terms applicable for US Federal Government officers or employees acting as part of their official duties; on a worldwide, perpetual, irrevocable, royalty-free basis to BMJ Publishing Group Ltd ("BMJ") its licensees and where the relevant Journal is co-owned by BMJ to the co-owners of the Journal, to publish the Work in this journal and any other BMJ products and to exploit all rights, as set out in our licence.

The Submitting Author accepts and understands that any supply made under these terms is made by BMJ to the Submitting Author unless you are acting as an employee on behalf of your employer or a postgraduate student of an affiliated institution which is paying any applicable article publishing charge ("APC") for Open Access articles. Where the Submitting Author wishes to make the Work available on an Open Access basis (and intends to pay the relevant APC), the terms of reuse of such Open Access shall be governed by a Creative Commons licence – details of these licences and which Creative Commons licence will apply to this Work are set out in our licence referred to above.

Other than as permitted in any relevant BMJ Author's Self Archiving Policies, I confirm this Work has not been accepted for publication elsewhere, is not being considered for publication elsewhere and does not duplicate material already published. I confirm all authors consent to publication of this Work and authorise the granting of this licence.

For peer review only

What is the impact of multimorbidity on the risk of hospitalization in older adults? A systematic review study protocol

Luciana Pereira Rodrigues[1]
Andréa Toledo de Oliveira Rezende[1]
Letícia de Almeida Nogueira e Moura[1]
Bruno Pereira Nunes[2]
Cesar de Oliveira[3]
Matias Noll[4,5]
Erika Aparecida Silveira[1,3,*]

[1] Graduate Program in Health Sciences, School of Medicine, Federal University of Goiás, Goiânia, Brazil
[2] Graduate Nursing Program, School of Nursing, Federal University of Pelotas, Pelotas, Brazil
[3] Department of Epidemiology & Public Health, Institute of Epidemiology & Health Care, University College London, London, UK
[4] Instituto Federal Goiano, Ceres, Brazil
[5] Department of Sports Science and Clinical Biomechanics, University of Southern Denmark, Odense, Denmark

Emails:
lucianarpereira7@gmail.com
rezende.andrea@hotmail.com
leticiaanm@gmail.com
matias.noll@ifgoiano.edu.br
c.oliveira@ucl.ac.uk
erikasil@terra.com.br
nunesbp@gmail.com

***Corresponding author:** Erika Aparecida Silveira
Graduate Program in Health Sciences, School of Medicine, Federal University of Goiás, Goiânia, Brazil, Post Code: 74605-050
Tel: +55 6232096146

For peer review only

E-mail: erikasil@terra.com.br

Abstract

Introduction The occurrence of multiple concomitant chronic diseases (multimorbidity) is increasing globally, along with the percentage of older adults affected by it. Multimorbidity is associated with the concomitant use of multiple medications, a greater possibility of adverse effects, and increased risk of hospitalization. Therefore, this systematic review study protocol aims to analyse the impact of multimorbidity on the incidence of hospitalization in older adults, and assess whether this impact changes according to factors such as gender, age, institutionalization, and socioeconomic status. This study will also review the average length of hospital stay and the incidence of hospital readmission.

Methods and analysis A systematic review of the literature will be carried out using the PubMed, Embase, and Scopus databases. The inclusion criteria will incorporate cross-sectional, cohort, and case-control studies that analysed the association between multimorbidity (defined as the presence of ≥ 2 and/or ≥ 3 chronic conditions and complex multimorbidity) and hospitalization (yes/no, days of hospitalization, and number of readmissions) in older adults (aged ≥ 60 years or > 65 years). Effect measures will be quantified, including odds ratios, prevalence ratios, and relative risk, along with their associated 95% confidence intervals. The aim of this study is to improve the treatment of older adults with multimorbidity and to avoid hospitalizations, thus contributing to improvements in public health care policy geared towards reducing hospitalization later in life and associated high healthcare costs and early mortality.

Ethics and dissemination Ethical approval is not required. The results will be disseminated via submission for publication to a peer-reviewed journal when complete. The results of this review may provide the basis for preventive actions seeking to reduce hospitalizations later in life and guide future research on the topic, identifying possible gaps and limitations of the existing literature.

PROSPERO registration number CRD42021229328

Article Summary Strengths of this study include the absence of restrictions in terms of language and year of publication, the use of methodological procedures that will assess the risk of bias in selecting studies, and inclusion of the three most commonly used definitions of multimorbidity. Potential limitations include the methodological diversity used in research involving multimorbidity, such as differences in the amount and type of chronic conditions included in the studies, and the stratification by age groups that may make it difficult to conduct a meta-analysis.

Introduction

Chronic non-communicable diseases (NCDs) are considerably increasing globally, particularly in low- and middle-income countries, due to lifestyle changes, accelerated urbanization, and increased longevity (1). Multimorbidity is defined as the co-occurrence of two or more chronic conditions in the same individual (2–6). Recent studies have also used the term 'complex multimorbidity', which is defined as 'the co-occurrence of three or more chronic conditions affecting three or more different body systems in one person, without defining an index chronic condition' (7).

Despite the growing research in the field, there are still some difficulties in relation to the specific definitions of multimorbidity and comorbidity (8–10). Comorbidity is defined as the co-occurrence of chronic conditions from an index disease (11). Multimorbidity, however, focuses on the interaction of several coexisting diseases and not on a predominant condition (12). Although the terms are similar, they should not be used interchangeably. In the present study, we will use the terms multimorbidity, which was incorporated into Medical Subject Headings (MeSH) in 2018, and complex multimorbidity (5,13).

The prevalence of multimorbidity has increased worldwide and affects over 50% of older adults, as increased life expectancy and longevity are associated with a greater disease burden (14,15). In this age group, the simultaneous occurrence of two or more chronic diseases is more common than in younger individuals, leading to the concomitant use of multiple medications and increasing the likelihood of adverse effects, mortality, and a higher number of hospitalizations (3,16–18).

For peer review only

The use of healthcare services, including hospitalization, is associated with specific factors such as sociodemographic variables, particularly socioeconomic status, and the co-occurrence of chronic conditions (19). In a study of sixteen European countries, multimorbidity was found to be associated with a greater number and length of hospitalization episodes in individuals aged 50 years or older (20). Another study conducted in southern Brazil found an association between multimorbidity and hospitalizations in individuals aged ≥ 60 years (21). As a result, some studies have found an association between multimorbidity and hospitalizations (20,21), whereas other studies have found an association only in participants over 80 years of age (22,23). Therefore, there is evidence to indicate that greater hospitalization rates are found in people with multimorbidity, however the evidence from previous studies varied and they were not standardized (24,25).

Multimorbidity negatively impacts on health and quality of life, and increases expenditure on healthcare (26,27). It is, therefore, crucial to obtain a deeper understanding of the impact of multimorbidity on hospitalizations later in life. This will potentially guide the development of public health policies and improve the care and treatment of older adults with multimorbidity. It will also lead to reductions in the number of hospitalization episodes and their associated negative consequences, such as loss of mobility (28), stress, risk of falling, poor mental health (29), cognitive impairment, social isolation, and decreased quality of life (30). Furthermore, older adults with multimorbidity use more health services, which is associated with an increased risk of death (31).

Despite the importance of the problem, only one systematic review on this topic (published in 2011) was identified (32). This systematic review focused on individuals with multiple chronic conditions and analysed several outcomes, including the use (number of medical consultations, use of medication, and use of hospital services) and cost of the health services. However, this systematic review did not provide further details on the impact of multimorbidity on hospitalizations in older adults. The present study aims to provide a better understanding of this topic. More importantly, many articles presenting new evidence on the subject have been published in the last 10 years.

The impact of multimorbidity on hospitalization episodes in older adults has not been previously investigated in a systematic review and meta-analysis. Therefore, the objective of this systematic review study protocol is to analyse the impact of

For peer review only

multimorbidity on the incidence of hospitalization in older adults and assess whether this impact varies by gender, age, institutionalization, and socioeconomic status. This study will also review the average length of hospital stay and the incidence of hospital readmission. The relevance of the topic for public healthcare and particularly geriatrics/gerontology relates to the increasing ageing population globally and its effect on society, particularly with regards to health (33).

Methods and analysis

This systematic review study will be conducted and reported following the *Preferred Reporting Items for Systematic Reviews and Meta-Analyses* (PRISMA) approach (34). The review will adopt the PECO structure (Population, Exposure, Comparator, and Outcome) recommended for systematic reviews (35). The PECO structure was defined as ‘P’ (elderly in the community), ‘E’ (multimorbidity), ‘C’ (associated factors), and ‘O’ (hospitalization). This systematic review protocol will be registered with the *International Prospective Register of Systematic Reviews* (PROSPERO). Any changes that occur during the study will be reported through the PROSPERO website and the final manuscript.

Search strategy and eligibility criteria

From December 2020 to February 2021, searches will be carried out in the PubMed, Embase, and Scopus databases by two independent researchers. There will be no restriction on the language and year of publication of the included studies. In the search strategy, MeSH terms and relevant keywords related to multimorbidity, hospitalization, and the elderly will be used, with the objective being to cover all articles on the topic (Table 1).

Table 1. Search strategy for studies on multimorbidity and hospitalization in older adults

1 = "multiple chronic conditions" OR multimorbidity OR multimorbidit* OR "multi morbidit*" OR multi-morbidity OR "chronic conditions multiple" OR "multiple chronic health conditions" OR "multiple chronic medical conditions" OR "multiple chronic illnesses" OR "chronic illnesses multiple" OR "multiple chronic diseases" OR multidisease OR multidiseases OR "multiple condition" OR "complex needs" OR "concurrent chronic conditions" OR "concurrent chronic diseases" OR "concurrent chronic disorders" OR "concurrent chronic health conditions" OR "concurrent chronic illnesses" OR "concurrent

For peer review only

chronic medical conditions" OR "multiple chronic disorders" OR "simultaneous chronic illnesses" OR "simultaneous chronic medical conditions"

2 = elderly OR elder OR aged OR ageing OR aging OR "old adults" OR "older adults" OR "older people" OR "old people" OR "geriatric" OR "aged patient" OR "aged people" OR "aged person" OR "aged subject" OR "elderly patient" OR "elderly people" OR "elderly person" OR "elderly subject" OR "senior citizen" OR senium

3 = hospitalization OR "patient readmission" OR inpatients OR hospitalized OR "health services" OR "medical assistance" OR "intensive care unity" OR "health care utilization" OR "length of stay" OR "short stay hospitalization" OR" hospital admission" OR "admission, hospital" OR "patient admission" OR "health care use" OR "health care utilisation" OR "health resource utilization" OR "health service use" OR "health service utilisation" OR "health service utilization" OR "health service utilization pattern" OR "health services use" OR "health services utilisation" OR "health services utilization" OR "utilization, health care" OR "hospital patient" OR "hospitalised patient" OR "hospitalised patients" OR "hospitalized patient" OR "hospitalized patients" OR "in-hospital patient" OR "in-hospital patients" OR "in-patient" OR "in-patients" OR inpatient OR "patient, hospital"

4 = 1 AND 2 AND 3

The inclusion criteria are: (a) cross-sectional, cohort, and case-control studies; (b) study subjects aged 60 years or older; (c) use of the definition of multimorbidity of ≥2 or ≥3 concomitant illnesses and complex multimorbidity; and (d) hospitalization as the outcome.

There are different ways to standardize and define multimorbidity. Despite the suggestion of Fortin et al. (36) to apply the definition as being the co-occurrence of three or more chronic conditions in the same individual, to better identify individuals who need more health care (as is often seen in the elderly population) the definition most used in the studies is the co-occurrence of two or more chronic conditions (27,36). In the studies, the difference in prevalence between ≥ 2 and ≥ 3 multimorbidities in general is 12.9% (15). Another proposed definition that aims to identify those individuals who need more complex health care is complex multimorbidity, defined as the ‘co-occurrence of three or more chronic conditions that affect three or more different body systems in a person, without defining a chronic index condition’ (7). Thus, this review will include studies that defined multimorbidity as ≥ 2 and ≥ 3 chronic conditions, and complex multimorbidity, in order to ensure a better methodological agreement and increase the possibility of conducting a meta-analysis.

For peer review only

Studies with incomplete data, review articles, ecological studies, case reports or case series, randomized clinical trials, and duplicate and unavailable data even after contacting the authors will be excluded. Studies addressing an index disease (such as cancer, heart disease, or depression) or another age group will also be excluded, unless the groups were stratified so that data on the elderly could be extracted and different outcomes could be analysed together (for example, hospitalization and functional capacity).

Training of the reviewers

The authors responsible for conducting the search and the inclusion/exclusion stage of the studies (LPR and ATOR) will be appropriately trained. The application of the eligibility criteria will be tested in 50 titles and abstracts of the articles obtained from the search strategy. Subsequently, the reviewers will also receive training to identify the risk of bias in the studies included. For this step, Mendeley and Rayyan software programs will be used (37) to manage references and control the procedures performed.

Review process

After searching the databases, duplicate studies will be deleted using Mendeley software. Two independent authors (LPR and ATOR) will then read the titles and abstracts of all articles selected in the database search process using Rayyan software. Disagreements will be discussed and resolved by a third reviewer (EAS).

Agreement between reviewers will be measured using Cohen's Kappa statistic (38), in which values closer to one (>0.6) indicate a higher level of agreement between reviewers. The studies selected in the earlier stages (title and abstract screening) will be read in full and evaluated according to the eligibility criteria. If any data that is important to the study is unavailable in the article, one of the researchers will directly contact the respective authors for clarification. After all of the above stages have taken place, those studies considered eligible will be included in the systematic review. A flowchart of the planned review process is shown in Figure 1.

For peer review only

Data extraction and evaluation of the study quality

Data will be extracted using a standardized form prepared by the authors, evaluating: author/year/location (city/country), type of study, target population (number of subjects, age group, gender, socioeconomic status/income of the country where the study was conducted or the income level of the elderly population, if analysed), the definition of multimorbidity used (≥2, ≥3, or complex multimorbidity; and the exact number and type of chronic conditions considered), occurrence of hospitalization (yes/no), and the number and days of hospitalizations and readmissions. The impact of multimorbidity on hospitalizations i.e. length and number, will be measured by prevalence ratios, incidence ratios, odds ratios, relative risk, and the respective 95% confidence intervals and *p*-values (Table 2). If necessary, additional columns will be added to the data extraction table.

The Downs & Black Scale will be used (39) to analyse the risk of bias during study selection. However, not all items on the list are applicable, with only 19 of them relevant: 1–3, 5–7, 9–12, 15–18, 20, 21, and 25–27. Those with a score higher than 70% will be considered as having a low risk of bias. The Grading of Recommendations, Assessment, Development, and Evaluation (GRADE) will be used to assess the quality of the evidence of the selected studies (40). The quality of evidence will be classified into four grades: high, moderate, low, or very low.

Statistical analysis

The proposed statistical analyses will be performed using Stata software, version 14.0. The outcome of this study will be the hospitalization of elderly people who have multimorbidity (≥2 or ≥3 chronic conditions and complex multimorbidity).

The possibility of a meta-analysis will be determined based on the homogeneity of the selected studies, using the fixed vs. random effects model. Heterogeneity will be assessed using the Chi-square test, with a significance level of $p < 0.05$. The I-square (I^2) statistic will be used to assess the magnitude of inconsistency, in which results greater than 75% indicate high heterogeneity, results between 25% to 75% indicating moderate heterogeneity, and results less than 25% indicate low heterogeneity (41–43).

For peer review only

Patient and Public Involvement

No patient involved.

Discussion

Studies conducted in older adults are of major importance, as the impact on health and the risks in this age group are different from those in the general population. Understanding the impact of multimorbidity on hospitalizations later in life will lead to improvements in treatment and the guidance provided by healthcare professionals, focusing on preventing hospitalizations as well as the development of public policies aimed at older adults with multimorbidity. The results of this study may also contribute to improving the management of national health agencies and private healthcare systems, thus contributing to a reduction of costs experienced by the healthcare system.

This systematic review study protocol describes the first study to investigate the impact of multimorbidity on hospitalizations in older adults as well as aspects related to this age group, particularly those aged 80 and older, differentiated in terms of gender and income of the population or income level of the country surveyed. This study will facilitate a deeper understanding of this topic in this age group and will support future research, reinforcing the importance of studies on this subject. Strengths of this study include the absence of restrictions in terms of language and year of publication, the use of methodological procedures that will assess the risk of bias in selecting studies, and inclusion of the three most commonly used definitions of multimorbidity. Potential limitations include the methodological diversity used in research involving multimorbidity, such as differences in the amount and type of chronic conditions included in the studies, and the stratification by age groups that may make it difficult to conduct a meta-analysis.

The results of this review may provide the basis for preventive actions seeking to reduce hospitalizations later in life and guide future research on the topic, identifying possible gaps and limitations of the existing literature.

For peer review only

Ethical aspects

Ethical approval was not required for this study, as the data used comprises of peer-reviewed publications and information that could identify the subjects of the original studies was not included.

References

1. Habib SH, Saha S. Burden of non-communicable disease: Global overview. Diabetes Metab Syndr Clin Res Rev [Internet]. 2010;4(1):41–7.
2. Almirall J, Fortin M. The coexistence of terms to describe the presence of multiple concurrent diseases. J Comorb. 2013;3:4–9.
3. Salive ME. Multimorbidity in older adults. Epidemiol Rev. 2013;35:75–83.
4. World Health Organization. Multimorbidity. Technical series on safer patient care. Geneva World Health Organization. 2016. Available from: https://apps.who.int/iris/bitstream/10665/252275/1/9789241511650-eng.pdf
5. Valderas JM, Starfield B, Sibbald B, Salisbury C, Roland M. Defining comorbidity: Implications for understanding health and health services. Ann Fam Med. 2009;7(4):357–63.
6. Johnston MC, Crilly M, Black C, Prescott GJ, Mercer SW. Defining and measuring multimorbidity: A systematic review of systematic reviews. Eur J Public Health. 2019;29(1):182–9.
7. Harrison C, Britt H, Miller G, Henderson J. Examining different measures of multimorbidity, using a large prospective cross-sectional study in Australian general practice. BMJ Open. 2014;4(7):e004694.
8. Nicholson K, Makovski TT, Griffith LE, Raina P, Stranges S, van den Akker M. Multimorbidity and comorbidity revisited: refining the concepts for international health research. J Clin Epidemiol. 2019;105:142–6.
9. Gulbech Ording A, Toft Sørensen H. Concepts of comorbidities, multiple morbidities, complications, and their clinical epidemiologic analogs. Clin Epidemiol. 2013;5(1):199–203.
10. Lefèvre T, d'Ivernois JF, De Andrade V, Crozet C, Lombrail P, Gagnayre R. What do we mean by multimorbidity? An analysis of the literature on multimorbidity measures, associated factors, and impact on health services organization. Rev

For peer review only

Epidemiol Sante Publique. 2014;62(5):305–14.

11. Feinstein AR. The pre-therapeutic classification of co-morbidity in chronic disease. J Chronic Dis. 1970;23(7):455–68.
12. Boeckxstaens P, Petrovic M. Multimorbidity: Definition, assessment, measurement and impact. In: Encyclopedia of Biomedical Gerontology. Elsevier Inc.; 2019. pp. 455–60.
13. Van Den Akker M, Buntinx F, Knottnerus JA. Comorbidity or multimorbidity: What's in a name? A review of literature. Eur J Gen Pract. 1996;2(2):65–70.
14. Marengoni A, Angleman S, Melis R, Mangialasche F, Karp A, Garmen A, et al. Aging with multimorbidity: A systematic review of the literature. Ageing Res Rev. 2011;10(4):430–9.
15. Nguyen H, Manolova G, Daskalopoulou C, Vitoratou S, Prince M, Prina AM. Prevalence of multimorbidity in community settings: A systematic review and meta-analysis of observational studies. 2019;9:1–15.
16. Boyd, Cynthia M; Fortin M. Future of multimorbidity research: How should understanding of multimorbidity inform health system design? Public Health Rev. 2010;32(2):451–74.
17. Barnett K, Mercer SW, Norbury M, Watt G, Wyke S, Guthrie B. Epidemiology of multimorbidity and implications for health care, research, and medical education: A cross-sectional study. Lancet. 2012;380(9836):37–43.
18. Reinhart L, Knight W, Roberts L, Mendes C. HHS Public Access Global Burden of Disease Study 2010. Lancet. 2012;380(9859):2163–96.
19. Andersen R, NewmaN JF. Societal and individual determinants of medical care utilization in the United States. Milbank Q. 2005;83(4):1–28.
20. Palladino R, Lee JT, Ashworth M, Triassi M, Millett C. Associations between multimorbidity, healthcare utilisation and health status: Evidence from 16 European countries. Age Ageing. 2016;45(3):431–5.
21. Nunes BP, Soares MU, Wachs LS, Volz PM, Saes M de O, Duro SMS, et al. Hospitalization in older adults: Association with multimorbidity, primary health care and private health plan. Rev Saúde Pública. 2017;51(43):1–9.
22. Dong H, Wressle E, Marcusson J. Factors associated with high use of health service among 85-year-old men and women: A Swedish population-based study. Eur Geriatr Med. 2012;3:S8.
23. Nägga K, Dong H-J, Marcusson J, Skoglund SO, Wressle E. Health-related factors

For peer review only

associated with hospitalization for old people: Comparisons of elderly aged 85 in a population cohort study. Arch Gerontol Geriatr. 2012;54(2):391–7.

24. Palladino R, Pennino F, Finbarr M, Millett C, Triassi M. Multimorbidity and health outcomes in older adults in ten European health systems, 2006–15. Health Aff. 2019;38(4):613–23.
25. Zulman DM, Chee CP, Wagner TH, Yoon J, Cohen DM, Holmes TH, et al. Multimorbidity and healthcare utilisation among high-cost patients in the US Veterans Affairs Health Care System. BMJ Open. 2015;5(4):1–9.
26. Palladino R, Lee JT, Ashworth M, Triassi M, Millett C. Associations between multimorbidity, healthcare utilisation and health status: evidence from 16 European countries. Age Ageing. 2016;45(3):431–5.
27. Glynn LG, Valderas JM, Healy P, Burke E, Newell J, Gillespie P, et al. The prevalence of multimorbidity in primary care and its effect on health care utilization and cost. Fam Pract. 2011;28(5):516–23.
28. The Academy of Medical Sciences. Multimorbidity: a priority for global health research. 2018. Available from: https://acmedsci.ac.uk/file-download/82222577
29. Carvalho TC, Valle AP do, Jacinto AF, Mayoural VF de S, Boas PJFV. Impacto da hospitalização na funcionalidade de idosos: Estudo de coorte. Rev Bras Geriatr Gerontol. 2018;21(2):134–42.
30. Rosa PH da, Beuter M, Benetti ERR, Bruinsma JL, Venturini L, Backes C. Stressors factors experienced by hospitalized elderly from the perspective of the Neuman Systems Model. Esc Anna Nery. 2018;22(4):1–9.
31. Cornette P, Swine C, Malhomme B, Gillet JB, Meert P, D'Hoore W. Early evaluation of the risk of functional decline following hospitalization of older patients: Development of a predictive tool. Eur J Public Health. 2006;16(2):203–8.
32. Pereira B, Ramos T, Iven G, Thumé E, Augusto L. Multimorbidity and mortality in older adults: A systematic review and. Arch Gerontol Geriatr. 2016;67:130–8.
33. Lehnert T, Heider D, Leicht H, Heinrich S, Corrieri S, Luppa M, et al. Review: Health care utilization and costs of elderly persons with multiple chronic conditions. Med Care Res Rev. 2011;68(4):387–420.
34. Chaimowicz F, Barcelos EM, Madureira MDS, Ribeiro MT de F. Saúde do idoso. 2013.
35. Moher D, Shamseer L, Clarke M, Ghersi D, Liberati A, Petticrew M, et al.

For peer review only

Preferred reporting items for systematic review and meta-analysis protocols (PRISMA-P) 2015 statement. Syst Rev. 2015;4(1):1–9.

36. Morgan RL, Whaley P, Thayer KA, Schünemann HJ. Identifying the PECO: A framework for formulating good questions to explore the association of environmental and other exposures with health outcomes. Environ Int. 2018;121:1027–31.
37. Fortin M, Stewart M, Poitras M, Maddocks H. A systematic review of prevalence studies on multimorbidity: Toward a more uniform methodology. Ann Fam Med. 2012;10(2):142–51.
38. Ouzzani M, Hammady H, Fedorowicz Z, Elmagarmid A. Rayyan-a web and mobile app for systematic reviews. Syst Rev. 2016;5(1):210.
39. Cohen J. Psychological Bulletin. Am Psychol Assoc. 1968;70(4):213–20.
40. Downs SH, Black N. The feasibility of creating a checklist for the assessment of the methodological quality both of randomised and non-randomised studies of health care interventions. J Epidemiol Community Health. 1998;52(6):377–84.
41. Guyatt G, Oxman AD., Akl EA, Kunz R, Vist G, Brozek J, et al. GRADE guidelines: 1. Introduction-GRADE evidence profiles and summary of findings tables. J Clin Epidemiol. 2011;64(4):383–94.
42. Higgins JPT, Thompson SG, Deeks JJ, Altman DG. Measuring inconsistency in meta-analyses. BMJ. 2003;327:557–60.
43. Brasil M da S. No Title. 2014th ed. Brasília - DF; 2014. 132 p. Available from: http://bvsms.saude.gov.br/bvs/publicacoes/diretrizes_metodologicas_estudos_acuracia_diagnostica.pdf
44. Rodrigues CL, Ziegelmann PK. Seção de bioestatística metanálise: Um guia prático meta-analysis: A practical guide. Rev HCPA. 2010;30:436–47.

Authors' contributions EAS, LPR, and ATOR formulated to the conception and design of the study; EAS, LPR, ATOR, MN and CO contributed to the methodologic aspects; EAS, LPR, ATOR, LANM, BPN, and MN wrote the protocol; EAS, CO, and MN carried out a final review of the protocol.

Acknowledgments We thank the Instituto Federal Goiano for its support and Capes for the master degree scholarship of L.P. Rodrigues.

Funding statement Dr Cesar de Oliveira is supported by the Economic and Social Research Council (grantES/T008822/1).

For peer review only

Competing interests statement The authors declare no conflicts of interest.

Table 2. Form for extracting data on the impact of multimorbidity on hospitalizations in the elderly.

Author Year Location	Type of study	Target population[a]	Multimorbidity[b]	Hospitalization[b]	Impact of multimorbidity on hospitalizations[c]	Impact of multimorbidity on hospitalizations[d]	Type of analysis performed and adjustments for potential confounders

[a]Institutionalized or from the community
[b]Definition, prevalence, and confidence interval of the prevalence
[c]In hospitalized subjects, by length of hospitalization and readmission (measure of odds ratio impact, prevalence ratio - with the respective confidence interval)
[d]According to gender, age group, and socioeconomic status

For peer review only

Figure 1. Flowchart of the selection criteria for the study

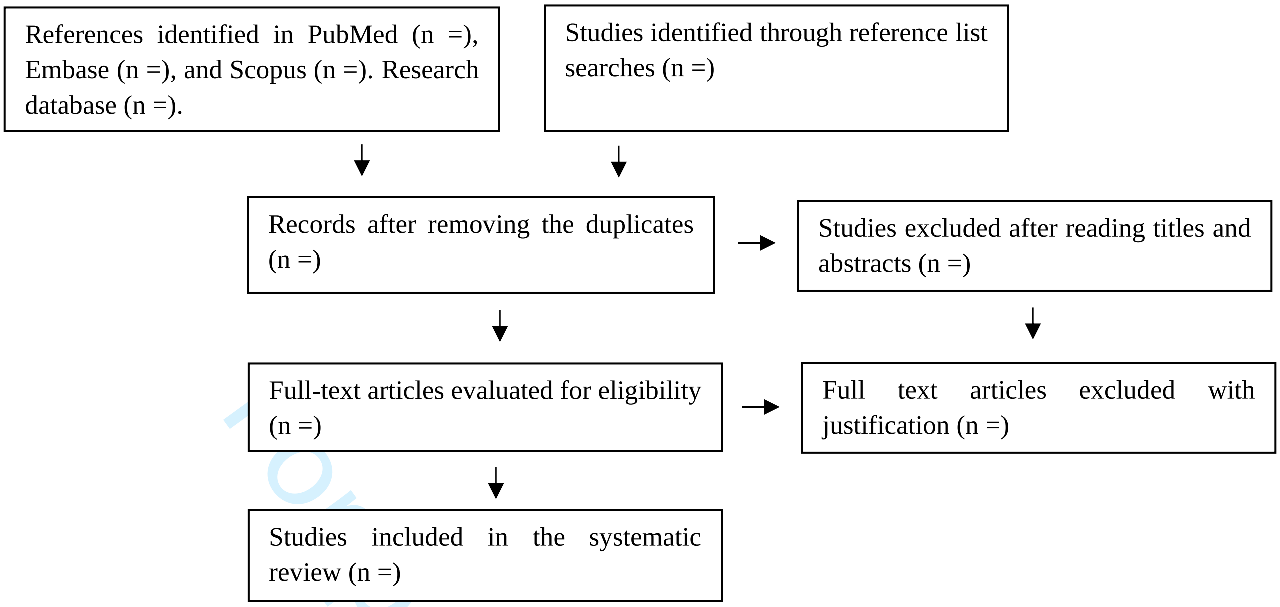

For peer review only

PRISMA-P 2015 Checklist

This checklist has been adapted for use with systematic review protocol submissions to BioMed Central journals from Table 3 in Moher D et al: Preferred reporting items for systematic review and meta-analysis protocols (PRISMA-P) 2015 statement. *Systematic Reviews* 2015 **4**:1

An Editorial from the Editors-in-Chief of *Systematic Reviews* details why this checklist was adapted - **Moher D, Stewart L & Shekelle P:** Implementing PRISMA-P: recommendations for prospective authors. *Systematic Reviews* 2016 **5**:15

Section/topic	#	Checklist item	Information reported		
			Yes	No	
**ADMINISTRATIVE INFORMATION**					
**Title**					
Identification	1a	Identify the report as a protocol of a systematic review	X		
Update	1b	If the protocol is for an update of a previous systematic review, identify as such		X	
**Registration**	2	If registered, provide the name of the registry (e.g., PROSPERO) and registration number in the Abstract	X		
**Authors**					
Contact	3a	Provide name, institutional affiliation, and e-mail address of all protocol authors; provide physical mailing address of corresponding author	X		
Contributions	3b	Describe contributions of protocol authors and identify the guarantor of the review	X		
**Amendments**	4	If the protocol represents an amendment of a previously completed or published protocol, identify as such and list changes; otherwise, state plan for documenting important protocol amendments		X	
**Support**					
Sources	5a	Indicate sources of financial or other support for the review	X		
Sponsor	5b	Provide name for the review funder and/or sponsor	X		
Role of sponsor/funder	5c	Describe roles of funder(s), sponsor(s), and/or institution(s), if any, in developing the protocol	X		
**INTRODUCTION**					
**Rationale**	6	Describe the rationale for the review in the context of what is already known	X		

For peer review only

For peer review only - http://bmjopen.bmj.com/site/about/guidelines.xhtml

BioMed Central The Open Access Publisher

Section/topic	#	Checklist item	Information reported		
			Yes	No	
**Objectives**	7	Provide an explicit statement of the question(s) the review will address with reference to participants, interventions, comparators, and outcomes (PICO)	X		
**METHODS**					
**Eligibility criteria**	8	Specify the study characteristics (e.g., PICO, study design, setting, time frame) and report characteristics (e.g., years considered, language, publication status) to be used as criteria for eligibility for the review	X		
**Information sources**	9	Describe all intended information sources (e.g., electronic databases, contact with study authors, trial registers, or other grey literature sources) with planned dates of coverage	X		
**Search strategy**	10	Present draft of search strategy to be used for at least one electronic database, including planned limits, such that it could be repeated	X		
STUDY RECORDS					
Data management	11a	Describe the mechanism(s) that will be used to manage records and data throughout the review	X		
Selection process	11b	State the process that will be used for selecting studies (e.g., two independent reviewers) through each phase of the review (i.e., screening, eligibility, and inclusion in meta-analysis)	X		
Data collection process	11c	Describe planned method of extracting data from reports (e.g., piloting forms, done independently, in duplicate), any processes for obtaining and confirming data from investigators	X		
**Data items**	12	List and define all variables for which data will be sought (e.g., PICO items, funding sources), any pre-planned data assumptions and simplifications	X		
**Outcomes and prioritization**	13	List and define all outcomes for which data will be sought, including prioritization of main and additional outcomes, with rationale	X		
**Risk of bias in individual studies**	14	Describe anticipated methods for assessing risk of bias of individual studies, including whether this will be done at the outcome or study level, or both; state how this information will be used in data synthesis	X		
DATA					
**Synthesis**	15a	Describe criteria under which study data will be quantitatively synthesized	X		
	15b	If data are appropriate for quantitative synthesis, describe planned summary measures, methods of handling data, and methods of combining data from studies, including any planned exploration of consistency (e.g., I^2, Kendall's tau)	X		
	15c	Describe any proposed additional analyses (e.g., sensitivity or subgroup analyses, meta-regression)	X		
	15d	If quantitative synthesis is not appropriate, describe the type of summary planned	X		

For peer review only

BioMed Central
The Open Access Publisher

Section/topic	#	Checklist item	Information reported Yes	No	
**Meta-bias(es)**	16	Specify any planned assessment of meta-bias(es) (e.g., publication bias across studies, selective reporting within studies)	X		
**Confidence in cumulative evidence**	17	Describe how the strength of the body of evidence will be assessed (e.g., GRADE)	X		

For peer review only

BioMed Central
The Open Access Publisher

BMJ Open

What is the impact of multimorbidity on the risk of hospitalization in older adults? A systematic review study protocol

Journal:	*BMJ Open*
Manuscript ID	bmjopen-2021-049974.R1
Article Type:	Protocol
Date Submitted by the Author:	09-May-2021
Complete List of Authors:	Rodrigues, Luciana; Universidade Federal de Goiás, Graduate Program in Health Sciences<br>Rezende, Andréa; Universidade Federal de Goias, Graduate Program in Health Sciences<br>Nogueira, Letícia; Universidade Federal de Goiás, Graduate Program in Health Sciences<br>Nunes, BP; Universidade Federal de Pelotas, Department of Nursing<br>Noll, Matias; Instituto Federal Goiano; University of Southern Denmark, Department of Sports Science and Clinical Biomechanics<br>de Oliveira, Cesar; University College London, Epidemiology and Public Health<br>Silveira, Erika; Universidade Federal de Goias, Faculdade de Medicina; University College London, Department of Epidemiology & Public Health
**Primary Subject Heading**:	Public health
Secondary Subject Heading:	Epidemiology, Public health
Keywords:	GERIATRIC MEDICINE, EPIDEMIOLOGY, PUBLIC HEALTH

SCHOLARONE™
Manuscripts

I, the Submitting Author has the right to grant and does grant on behalf of all authors of the Work (as defined in the below author licence), an exclusive licence and/or a non-exclusive licence for contributions from authors who are: i) UK Crown employees; ii) where BMJ has agreed a CC-BY licence shall apply, and/or iii) in accordance with the terms applicable for US Federal Government officers or employees acting as part of their official duties; on a worldwide, perpetual, irrevocable, royalty-free basis to BMJ Publishing Group Ltd ("BMJ") its licensees and where the relevant Journal is co-owned by BMJ to the co-owners of the Journal, to publish the Work in this journal and any other BMJ products and to exploit all rights, as set out in our licence.

The Submitting Author accepts and understands that any supply made under these terms is made by BMJ to the Submitting Author unless you are acting as an employee on behalf of your employer or a postgraduate student of an affiliated institution which is paying any applicable article publishing charge ("APC") for Open Access articles. Where the Submitting Author wishes to make the Work available on an Open Access basis (and intends to pay the relevant APC), the terms of reuse of such Open Access shall be governed by a Creative Commons licence – details of these licences and which Creative Commons licence will apply to this Work are set out in our licence referred to above.

Other than as permitted in any relevant BMJ Author's Self Archiving Policies, I confirm this Work has not been accepted for publication elsewhere, is not being considered for publication elsewhere and does not duplicate material already published. I confirm all authors consent to publication of this Work and authorise the granting of this licence.

For peer review only

What is the impact of multimorbidity on the risk of hospitalization in older adults? A systematic review study protocol

Luciana Pereira Rodrigues[1]

Andréa Toledo de Oliveira Rezende[1]

Letícia de Almeida Nogueira e Moura[1]

Bruno Pereira Nunes[2]

Matias Noll[3,4]

Cesar de Oliveira[5]

Erika Aparecida Silveira[1,5*]

[1] Graduate Program in Health Sciences, School of Medicine, Federal University of Goiás, Goiânia, Brazil

[2] Graduate Nursing Program, School of Nursing, Federal University of Pelotas, Pelotas, Brazil

[3] Federal Institute of Goiania, Ceres, Brazil

[4] Department of Sports Science and Clinical Biomechanics, University of Southern Denmark, Odense, Denmark

[5]Department of Epidemiology & Public Health, Institute of Epidemiology & Health Care, University College London, London, UK

E-mails:

lucianarpereira7@gmail.com

rezende.andrea@hotmail.com

leticiaanm@gmail.com

nunesbp@gmail.com

matias.noll@ifgoiano.edu.br

c.oliveira@ucl.ac.uk

erikasil@terra.com.br

***Corresponding author:** Erika Aparecida Silveira

Graduate Program in Health Sciences, School of Medicine, Federal University of Goiás, Goiânia, Brazil, Post Code: 74605-050

For peer review only

Telephone: +55 6232096146; E-mail: erikasil@terra.com.br

Abstract

Introduction The development of multiple coexisting chronic diseases (multimorbidity) is increasing globally, along with the percentage of older adults affected by it. Multimorbidity is associated with the concomitant use of multiple medications, a greater possibility of adverse effects, and increased risk of hospitalization. Therefore, this systematic review study protocol aims to analyse the impact of multimorbidity on the occurrence of hospitalization in older adults and assess whether this impact changes according to factors such as sex, age, institutionalization, and socioeconomic status. This study will also review the average length of hospital stay and the occurrence of hospital readmission.

Methods and analysis A systematic review of the literature will be carried out using the PubMed, Embase, and Scopus databases. The inclusion criteria will incorporate cross-sectional, cohort, and case-control studies that analysed the association between multimorbidity (defined as the presence of ≥ 2 and/or ≥ 3 chronic conditions and complex multimorbidity) and hospitalization (yes/no, days of hospitalization, and number of readmissions) in older adults (aged ≥ 60 years or > 65 years). Effect measures will be quantified, including odds ratios, prevalence ratios, hazard ratios and relative risk, along with their associated 95% confidence intervals. The overall aim of this study is to widen knowledge and to raise reflections about the association between multimorbidity and hospitalization in older adults. Ultimately, its findings may contribute to improvements in public health policies resulting in cost reductions across healthcare systems.

Ethics and dissemination Ethical approval is not required. The results will be disseminated via submission for publication to a peer-reviewed journal when complete.

PROSPERO registration number CRD42021229328

For peer review only

Strengths and limitations of this study

- This review will not present restrictions in terms of language and year of publication regarding the articles that will be included in the search.
- The review process for selection and inclusion of the articles will be performed separately by two researchers and any disagreements will be mediated by a third reviewer in order to assure a consistent and strict application of the inclusion and exclusion criteria.
- Another strength is that there is no registry of previews systematic reviews on the association between multimorbidity and hospitalization focused on older adults.
- Additionally, this study will include the three most commonly used definitions of multimorbidity in order to reach a greater number of studies related to the theme.
- The limitation of this review might be the difficulty to conduct a meta-analysis since the potential selected studies may have used different definitions for multimorbidity as well as subgroups analysis stratified by age and sex.

Introduction

Chronic non-communicable diseases (NCDs) are considerably increasing globally, particularly in low- and middle-income countries, due to lifestyle changes, accelerated urbanization, and increased longevity (1). Multimorbidity is defined as the co-occurrence of two or more chronic conditions in the same individual (2–6). Recent studies have also used the term 'complex multimorbidity', which is defined as 'the co-occurrence of three or more chronic conditions affecting three or more different body systems in one person, without defining an index chronic condition' (7).

Despite the growing research in the field, there are still some difficulties in relation to the specific definitions of multimorbidity and comorbidity (8–10). Comorbidity is defined as the co-occurrence of chronic conditions from an index disease (11). Multimorbidity, however, focuses on the interaction of several coexisting diseases and not on a predominant condition (12). Although the terms are similar, they should not be used interchangeably. In the present study, we will use the terms multimorbidity, which was incorporated into Medical Subject Headings (MeSH) in 2018, and complex multimorbidity (5,13).

For peer review only

The prevalence of multimorbidity has increased worldwide and affects over 50% of older adults, as increased life expectancy and longevity are associated with a greater disease burden (14,15). In this age group, the simultaneous occurrence of two or more chronic diseases is more common than in younger individuals, leading to the associated use of multiple medications and increasing the likelihood of adverse effects, mortality, and a higher number of hospitalizations (3,16–18).

The use of healthcare services, including hospitalization, is associated with specific factors such as sociodemographic variables, particularly socioeconomic status, and the co-occurrence of chronic conditions (19). In a study of sixteen European countries, multimorbidity was found to be associated with a greater number and length of hospitalization episodes in individuals aged 50 years or older (20). Another study conducted in southern Brazil found an association between multimorbidity and hospitalizations in individuals aged ≥ 60 years (21). As a result, some studies have found an association between multimorbidity and hospitalizations (20,21), whereas other studies have found an association only in participants over 80 years of age (22,23). Therefore, there is evidence to indicate that greater hospitalization rates are found in people with multimorbidity, however the evidence from previous studies varied and they were not standardized (24,25).

Multimorbidity negatively impacts on health and quality of life, and increases expenditure on healthcare (26,27). It is, therefore, crucial to obtain a deeper understanding of the impact of multimorbidity on hospitalizations later in life. This will potentially guide the development of public health policies and improve the care and treatment of older adults with multimorbidity. It will also lead to reductions in the number of hospitalization episodes and their associated negative consequences, such as loss of mobility (28), stress, risk of falling, poor mental health (29), cognitive impairment, social isolation, and decreased quality of life (30). Furthermore, older adults with multimorbidity use more health services, which is associated with an increased risk of death (31).

Despite the importance of the problem, only one systematic review on this topic (published in 2011) was identified (32). This systematic review focused on individuals with multiple chronic conditions and analysed several outcomes, including the use (number of medical consultations, use of medication, and use of hospital services) and cost of the health services. However, this systematic review did not provide further details

For peer review only

on the impact of multimorbidity on hospitalizations in older adults. The present study aims to provide a better understanding of this topic. More importantly, many articles presenting new evidence on the subject have been published in the last 10 years.

The impact of multimorbidity on hospitalization episodes in older adults has not been previously investigated in a systematic review and meta-analysis. Therefore, the objective of this systematic review study protocol is to analyse the impact of multimorbidity on the occurrence of hospitalization in older adults and assess whether this impact varies by sex, age, institutionalization, and socioeconomic status. This study will also review the average length of hospital stay and the occurrence of hospital readmission. The relevance of the topic for public healthcare and particularly geriatrics/gerontology relates to the increasing ageing population globally and its effect on society, particularly with regards to health (33).

Methods and analysis

This systematic review study will be conducted and reported following the *Preferred Reporting Items for Systematic Reviews and Meta-Analyses* (PRISMA) approach (34). The review will adopt the PECO structure (Population, Exposure, Comparator, and Outcome) recommended for systematic reviews (35). The PECO structure was defined as 'P' (older adults in the community), 'E' (multimorbidity), 'C' (associated factors), and 'O' (hospitalization). This systematic review protocol was registered with the *International Prospective Register of Systematic Reviews* (PROSPERO) under the number CRD42021229328. Any changes that occur during the study will be reported through the PROSPERO website and the final manuscript.

Search strategy and eligibility criteria

From February 2021 to April 2021, searches will be carried out in the PubMed, Embase, and Scopus databases by two independent researchers. There will be no restriction on the language and year of publication of the included studies and articles published until 30 April 2021 will be considered. In the search strategy, MeSH terms and relevant keywords related to multimorbidity, hospitalization, and older adults will be used, with the objective being to cover all articles on the topic (Table 1).

Table 1. Search strategy for studies on multimorbidity and hospitalization in older adults

For peer review only

1 = "multiple chronic conditions" OR multimorbidity OR multimorbidit* OR "multi morbidit*" OR multi-morbidity OR "chronic conditions multiple" OR "multiple chronic health conditions" OR "multiple chronic medical conditions" OR "multiple chronic illnesses" OR "chronic illnesses multiple" OR "multiple chronic diseases" OR multidisease OR multidiseases OR "multiple condition" OR "complex needs" OR "concurrent chronic conditions" OR "concurrent chronic diseases" OR "concurrent chronic disorders" OR "concurrent chronic health conditions" OR "concurrent chronic illnesses" OR "concurrent chronic medical conditions" OR "multiple chronic disorders" OR "simultaneous chronic illnesses" OR "simultaneous chronic medical conditions"

2 = elderly OR elder OR aged OR ageing OR aging OR "old adults" OR "older adults" OR "older people" OR "old people" OR "geriatric" OR "aged patient" OR "aged people" OR "aged person" OR "aged subject" OR "elderly patient" OR "elderly people" OR "elderly person" OR "elderly subject" OR "senior citizen" OR senium

3 = hospitalization OR "patient readmission" OR inpatients OR hospitalized OR "health services" OR "medical assistance" OR "intensive care unity" OR "health care utilization" OR "length of stay" OR "short stay hospitalization" OR" hospital admission" OR "admission, hospital" OR "patient admission" OR "health care use" OR "health care utilisation" OR "health resource utilization" OR "health service use" OR "health service utilisation" OR "health service utilization" OR "health service utilization pattern" OR "health services use" OR "health services utilisation" OR "health services utilization" OR "utilization, health care" OR "hospital patient" OR "hospitalised patient" OR "hospitalised patients" OR "hospitalized patient" OR "hospitalized patients" OR "in-hospital patient" OR "in-hospital patients" OR "in-patient" OR "in-patients" OR inpatient OR "patient, hospital"

4 = 1 AND 2 AND 3

The inclusion criteria are: (a) cross-sectional, cohort, and case-control studies; (b) study subjects aged 60 years or older; (c) use of the definition of multimorbidity of ≥2 or ≥3 coexisting illnesses and complex multimorbidity; and (d) hospitalization as the outcome.

There are different ways to standardize and define multimorbidity. Despite the suggestion of Fortin et al. (36) to apply the definition as being the co-occurrence of three or more chronic conditions in the same individual, to better identify individuals who need more health care (as is often seen in older adults) the definition most used in the studies is the co-occurrence of two or more chronic conditions (27,36). In the studies, the difference in prevalence between ≥ 2 and ≥ 3 multimorbidities in general is 12.9% (15). Another proposed definition that aims to identify those individuals who need more complex health care is complex multimorbidity, defined as the 'co-occurrence of three or

For peer review only

more chronic conditions that affect three or more different body systems in a person, without defining a chronic index condition' (7). Thus, this review will include studies that defined multimorbidity as ≥ 2 and ≥ 3 chronic conditions, and complex multimorbidity, in order to ensure a better methodological agreement and increase the possibility of conducting a meta-analysis.

Studies with incomplete data, review articles, ecological studies, case reports or case series, randomized clinical trials, and duplicate and unavailable data even after contacting the authors will be excluded. Studies addressing an index disease (such as cancer, heart disease, or depression) or another age group will also be excluded, unless the groups were stratified so that data on older adults could be extracted and different outcomes could be analysed together (for example, hospitalization and functional capacity).

Training of the reviewers

The authors responsible for conducting the search and the inclusion/exclusion stage of the studies (LPR and ATOR) will be appropriately trained. The application of the eligibility criteria will be tested in 50 titles and abstracts of the articles obtained from the search strategy. Subsequently, the reviewers will also receive training to identify the risk of bias in the studies included. For this step, Mendeley and Rayyan software programs will be used (37) to manage references and control the procedures performed.

Review process

After searching the databases, duplicate studies will be deleted using Mendeley software. Two independent authors (LPR and ATOR) will then read the titles and abstracts of all articles selected in the database search process using Rayyan software. Disagreements will be discussed and resolved by a third reviewer (EAS).

Agreement between reviewers will be measured using Cohen's Kappa statistic (38), in which values closer to one (>0.6) indicate a higher level of agreement between reviewers. The studies selected in the earlier stages (title and abstract screening) will also be read in full and evaluated according to the eligibility criteria by two independents authors (LPR and ATOR). If any data that is important to the study is unavailable in the article, one of the researchers will directly contact the respective authors for clarification.

For peer review only

After all of the above stages have taken place, those studies considered eligible will be included in the systematic review. A flow chart of the planned review process is shown in Figure 1.

Data extraction and evaluation of the study quality

Data will be extracted using a standardized form prepared by the authors, evaluating: author/year/location (city/country), type of study, target population (number of subjects, age group, sex, socioeconomic status/income of the country where the study was conducted or the income level of older adults, if analysed), the definition of multimorbidity used (≥2, ≥3, or complex multimorbidity; and the exact number and type of chronic conditions considered), multimorbidity assessment method (data source and multimorbidity measures used to assess chronic disease), co-occurrence of hospitalization (yes/no), and the number and days of hospitalizations and readmissions. The impact of multimorbidity on hospitalizations i.e. length and number, will be measured by prevalence ratios, incidence ratios, odds ratios, hazard ratios, relative risk, and the respective 95% confidence intervals and *p*-values (Table 2). If necessary, additional columns will be added to the data extraction table.

The Downs & Black Scale will be used (39) to analyse the risk of bias during study selection. However, not all items on the list are applicable, with only 19 of them relevant: 1–3, 5–7, 9–12, 15–18, 20, 21, and 25–27. Those with a score higher than 70% will be considered as having a low risk of bias. The Grading of Recommendations, Assessment, Development, and Evaluation (GRADE) will be used to assess the quality of the evidence of the selected studies (40). The quality of evidence will be classified into four grades: high, moderate, low, or very low.

Statistical analysis

The evidence on the impact of multimorbidity in the occurrence of hospitalization in older adults will be summarized. In addition, a meta-analysis of the mean length of stay and the occurrence of hospital readmission will be carried out. Forest plot of random effects model will be built using odds ratios (OR) and their respective 95% confidence intervals (CI), both for the meta-analysis of multimorbidity impact in the occurrence of hospitalization and for analyzing the occurrence of hospital readmission. Relative risk

For peer review only

(RR), prevalence ratios and hazard ratios and their respective 95% confidence intervals (CI) will be converted into ORs and included in the analysis. The combined results will be stratified according to socioeconomic status, institutionalization, age and sex. The proposed statistical analysis will be performed using Stata14® SE (Stata Corp, College Station, TX, USA).

The heterogeneity will be quantified using I^2 (41) and a forest graphic will graphically display the effect sizes among studies (42). For the studies with I^2 values greater than or equal 50% (43), meta-regression may be performed according to the exposure variables described above. Publication bias will be assessed through a funnel plot (44) and asymmetry of the funnel plot will be evaluated using the Egger test (45).

Patient and Public Involvement

No patient involved.

Discussion

Studies conducted in older adults are of major importance, as the impact on health and the risks in this age group are different from those in the general population. Understanding the impact of multimorbidity on hospitalizations later in life will provide guidance to healthcare professionals, as well as the development of public policies aimed at older adults with multimorbidity. The results of this study may also contribute to improving the management of national health agencies and private healthcare systems, thus contributing to a reduction of costs experienced by the healthcare system.

This systematic review study protocol describes the first study to investigate the impact of multimorbidity on hospitalizations in older adults as well as aspects related to this age group, particularly those aged 80 and older, differentiated in terms of sex and income of the population or income level of the country surveyed. This study will facilitate a deeper understanding of this topic in this age group and will support future research, reinforcing the importance of studies on this subject. Strengths of this study include the absence of restrictions in terms of language and year of publication, the use of methodological procedures that will assess the risk of bias in selecting studies, and inclusion of the three most commonly used definitions of multimorbidity. Potential

For peer review only

limitations include the methodological diversity used in research involving multimorbidity, such as differences in the amount and type of chronic conditions included in the studies, and the stratification by age groups that may make it difficult to conduct a meta-analysis.

The results of this review may provide the basis for widening knowledge and to increase awareness of the main issues the association between multimorbidity and hospitalization in older adults. Our findings may also contribute to improvements in public health policies resulting in cost reductions across healthcare systems. Ultimately, this review will seek to guide future research on the topic, identifying possible gaps and limitations of the existing literature.

Ethical aspects

Ethical approval was not required for this study, as the data used comprises of peer-reviewed publications and information that could identify the subjects of the original studies was not included.

References

1. Habib SH, Saha S. Burden of non-communicable disease: Global overview. Diabetes Metab Syndr Clin Res Rev [Internet]. 2010;4(1):41–7.
2. Almirall J, Fortin M. The coexistence of terms to describe the presence of multiple concurrent diseases. J Comorb. 2013;3:4–9.
3. Salive ME. Multimorbidity in older adults. Epidemiol Rev. 2013;35:75–83.
4. World Health Organization. Multimorbidity. Technical series on safer patient care. Geneva World Health Organization. 2016. Available from: https://apps.who.int/iris/bitstream/10665/252275/1/9789241511650-eng.pdf
5. Valderas JM, Starfield B, Sibbald B, Salisbury C, Roland M. Defining comorbidity: Implications for understanding health and health services. Ann Fam Med. 2009;7(4):357–63.
6. Johnston MC, Crilly M, Black C, Prescott GJ, Mercer SW. Defining and measuring multimorbidity: A systematic review of systematic reviews. Eur J Public Health.

For peer review only

2019;29(1):182–9.

7. Harrison C, Britt H, Miller G, Henderson J. Examining different measures of multimorbidity, using a large prospective cross-sectional study in Australian general practice. BMJ Open. 2014;4(7):e004694.

8. Nicholson K, Makovski TT, Griffith LE, Raina P, Stranges S, van den Akker M. Multimorbidity and comorbidity revisited: refining the concepts for international health research. J Clin Epidemiol. 2019;105:142–6.

9. Gulbech Ording A, Toft Sørensen H. Concepts of comorbidities, multiple morbidities, complications, and their clinical epidemiologic analogs. Clin Epidemiol. 2013;5(1):199–203.

10. Lefèvre T, d'Ivernois JF, De Andrade V, Crozet C, Lombrail P, Gagnayre R. What do we mean by multimorbidity? An analysis of the literature on multimorbidity measures, associated factors, and impact on health services organization. Rev Epidemiol Sante Publique. 2014;62(5):305–14.

11. Feinstein AR. The pre-therapeutic classification of co-morbidity in chronic disease. J Chronic Dis. 1970;23(7):455–68.

12. Boeckxstaens P, Petrovic M. Multimorbidity: Definition, assessment, measurement and impact. In: Encyclopedia of Biomedical Gerontology. Elsevier Inc.; 2019. pp. 455–60.

13. Van Den Akker M, Buntinx F, Knottnerus JA. Comorbidity or multimorbidity: What's in a name? A review of literature. Eur J Gen Pract. 1996;2(2):65–70.

14. Marengoni A, Angleman S, Melis R, Mangialasche F, Karp A, Garmen A, et al. Aging with multimorbidity: A systematic review of the literature. Ageing Res Rev. 2011;10(4):430–9.

15. Nguyen H, Manolova G, Daskalopoulou C, Vitoratou S, Prince M, Prina AM. Prevalence of multimorbidity in community settings: A systematic review and meta-analysis of observational studies. 2019;9:1–15.

16. Boyd, Cynthia M; Fortin M. Future of multimorbidity research: How should understanding of multimorbidity inform health system design? Public Health Rev. 2010;32(2):451–74.

17. Barnett K, Mercer SW, Norbury M, Watt G, Wyke S, Guthrie B. Epidemiology of multimorbidity and implications for health care, research, and medical education: A cross-sectional study. Lancet. 2012;380(9836):37–43.

18. Reinhart L, Knight W, Roberts L, Mendes C. HHS Public Access Global Burden

For peer review only

of Disease Study 2010. Lancet. 2012;380(9859):2163–96.

19. Andersen R, NewmaN JF. Societal and individual determinants of medical care utilization in the United States. Milbank Q. 2005;83(4):1–28.

20. Palladino R, Lee JT, Ashworth M, Triassi M, Millett C. Associations between multimorbidity, healthcare utilisation and health status: Evidence from 16 European countries. Age Ageing. 2016;45(3):431–5.

21. Nunes BP, Soares MU, Wachs LS, Volz PM, Saes M de O, Duro SMS, et al. Hospitalization in older adults: Association with multimorbidity, primary health care and private health plan. Rev Saúde Pública. 2017;51(43):1–9.

22. Dong H, Wressle E, Marcusson J. Factors associated with high use of health service among 85-year-old men and women: A Swedish population-based study. Eur Geriatr Med. 2012;3:S8.

23. Nägga K, Dong H-J, Marcusson J, Skoglund SO, Wressle E. Health-related factors associated with hospitalization for old people: Comparisons of elderly aged 85 in a population cohort study. Arch Gerontol Geriatr. 2012;54(2):391–7.

24. Palladino R, Pennino F, Finbarr M, Millett C, Triassi M. Multimorbidity and health outcomes in older adults in ten European health systems, 2006–15. Health Aff. 2019;38(4):613–23.

25. Zulman DM, Chee CP, Wagner TH, Yoon J, Cohen DM, Holmes TH, et al. Multimorbidity and healthcare utilisation among high-cost patients in the US Veterans Affairs Health Care System. BMJ Open. 2015;5(4):1–9.

26. Palladino R, Lee JT, Ashworth M, Triassi M, Millett C. Associations between multimorbidity, healthcare utilisation and health status: evidence from 16 European countries. Age Ageing. 2016;45(3):431–5.

27. Glynn LG, Valderas JM, Healy P, Burke E, Newell J, Gillespie P, et al. The prevalence of multimorbidity in primary care and its effect on health care utilization and cost. Fam Pract. 2011;28(5):516–23.

28. The Academy of Medical Sciences. Multimorbidity: a priority for global health research. 2018. Available from: https://acmedsci.ac.uk/file-download/82222577

29. Carvalho TC, Valle AP do, Jacinto AF, Mayoural VF de S, Boas PJFV. Impacto da hospitalização na funcionalidade de idosos: Estudo de coorte. Rev Bras Geriatr Gerontol. 2018;21(2):134–42.

30. Rosa PH da, Beuter M, Benetti ERR, Bruinsma JL, Venturini L, Backes C. Stressors factors experienced by hospitalized elderly from the perspective of the

For peer review only

Neuman Systems Model. Esc Anna Nery. 2018;22(4):1–9.

31. Cornette P, Swine C, Malhomme B, Gillet JB, Meert P, D'Hoore W. Early evaluation of the risk of functional decline following hospitalization of older patients: Development of a predictive tool. Eur J Public Health. 2006;16(2):203–8.

32. Pereira B, Ramos T, Iven G, Thumé E, Augusto L. Multimorbidity and mortality in older adults: A systematic review and. Arch Gerontol Geriatr. 2016;67:130–8.

33. Lehnert T, Heider D, Leicht H, Heinrich S, Corrieri S, Luppa M, et al. Review: Health care utilization and costs of elderly persons with multiple chronic conditions. Med Care Res Rev. 2011;68(4):387–420.

34. Chaimowicz F, Barcelos EM, Madureira MDS, Ribeiro MT de F. Saúde do idoso. 2013.

35. Moher D, Shamseer L, Clarke M, Ghersi D, Liberati A, Petticrew M, et al. Preferred reporting items for systematic review and meta-analysis protocols (PRISMA-P) 2015 statement. Syst Rev. 2015;4(1):1–9.

36. Morgan RL, Whaley P, Thayer KA, Schünemann HJ. Identifying the PECO: A framework for formulating good questions to explore the association of environmental and other exposures with health outcomes. Environ Int. 2018;121:1027–31.

37. Fortin M, Stewart M, Poitras M, Maddocks H. A systematic review of prevalence studies on multimorbidity: Toward a more uniform methodology. Ann Fam Med. 2012;10(2):142–51.

38. Ouzzani M, Hammady H, Fedorowicz Z, Elmagarmid A. Rayyan-a web and mobile app for systematic reviews. Syst Rev. 2016;5(1):210.

39. Cohen J. Psychological Bulletin. Am Psychol Assoc. 1968;70(4):213–20.

40. Downs SH, Black N. The feasibility of creating a checklist for the assessment of the methodological quality both of randomised and non-randomised studies of health care interventions. J Epidemiol Community Health. 1998;52(6):377–84.

41. Higgins JPT, Thompson SG. Quantifying heterogeneity in a meta-analysis. Stat Med. 2002;21(11):1539–58. https://doi.org/10.1002/sim.1186.

42. Kang EY, Park Y, Li X, et al. ForestPMPlot: A flexible tool for visualizing heterogeneity between studies in meta-analysis. G3 Bethesda Md 2016;6(7):1793–1798.

43. Duval S, Tweedie R. Trim and fill: a simple funnel-plot-based method of testing

For peer review only

and adjusting for publication bias in meta-analysis. Biometrics. 2000;56(2):455–63. https://doi.org/10.1111/j.0006-341X.2000.00455.x.

44. Egger M, Davey Smith G, Schneider M, et al. Bias in meta-analysis detected by a simple, graphical test BMJ.1997;315(7109):629–34. https://doi.org/10.1136/bmj.315.7109.629.

45. Higgins JPT, Thompson SG, Deeks JJ, Altman DG. Measuring inconsistency in meta-analyses. BMJ. 2003;327:557–60.

Authors' contributions EAS, LPR, and ATOR formulated to the conception and design of the study; EAS, LPR, ATOR, MN and CO contributed to the methodologic aspects; EAS, LPR, ATOR, LANM, BPN, and MN wrote the protocol; EAS, CO, and MN carried out a final review of the protocol.

Acknowledgments We thank the Instituto Federal Goiano for its support and Capes for the master degree scholarship of L.P. Rodrigues.

Funding statement Dr Cesar de Oliveira is supported by the Economic and Social Research Council (grantES/T008822/1).

Competing interests statement The authors declare no conflicts of interest.

Table 2. Form for extracting data on the impact of multimorbidity on hospitalizations in older adults.

Author Year Location	Type of study	Target population[a]	Multimorbidity[b]	Multimorbidity assessment method[c]	Hospitalization[b]	Impact of multimorbidity on hospitalizations[d]	Impact of multimorbidity on hospitalizations[e]	Type of analysis performed and adjustments for potential confounders

[a]Institutionalized or from the community
[b]Definition, prevalence, and confidence interval of the prevalence
[c]Data source and multimorbidity measures used to investigate chronic disease (simple list of diseases, pre-compiled list of conditions or ad hoc tools).
[d]In hospitalized subjects, by length of hospitalization and readmission (measure of odds ratio impact, prevalence ratio, hazard ratio - with the respective confidence interval)
[e]According to sex, age group, and socioeconomic status

For peer review only

Figure 1. Preferred Reporting Items for Systematic Reviews and Meta-Analyses flow chart: search and inclusion process of studies to include in systematic review of the multimorbidity impact on the risk of hospitalization in older adults.

For peer review only

Figure 1. Preferred Reporting Items for Systematic Reviews and Meta-Analyses flow chart: search and inclusion process of studies to include in systematic review of the multimorbidity impact on the risk of hospitalization in older adults.

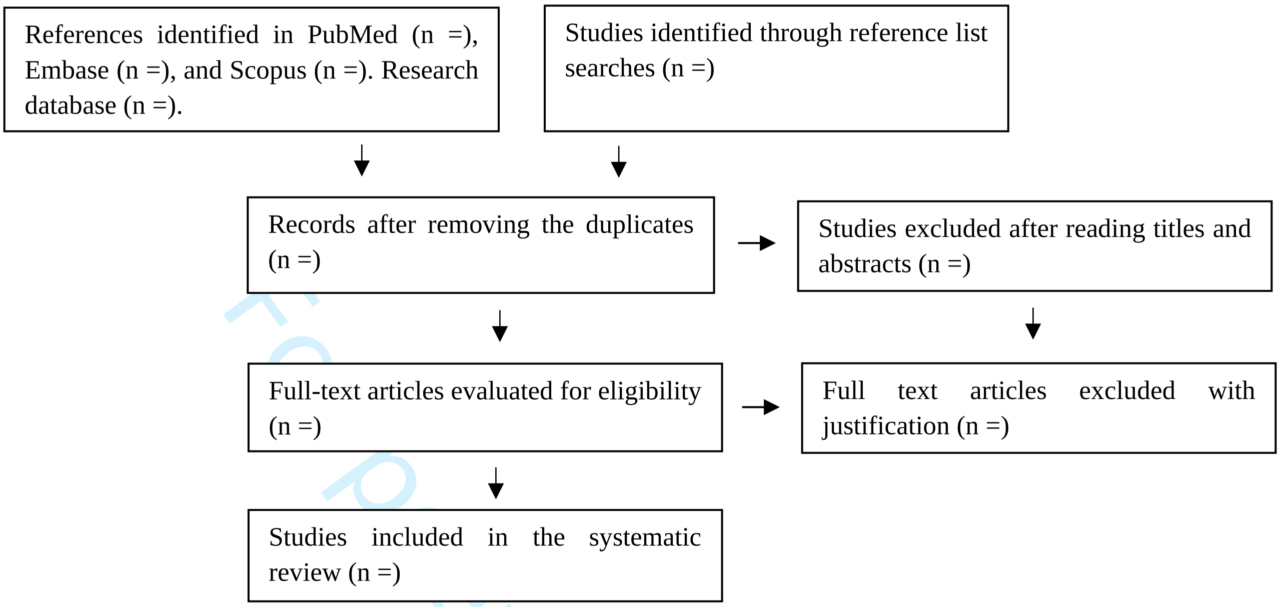

For peer review only

PRISMA-P 2015 Checklist

This checklist has been adapted for use with systematic review protocol submissions to BioMed Central journals from Table 3 in Moher D et al: Preferred reporting items for systematic review and meta-analysis protocols (PRISMA-P) 2015 statement. *Systematic Reviews* 2015 **4**:1

An Editorial from the Editors-in-Chief of *Systematic Reviews* details why this checklist was adapted - **Moher D, Stewart L & Shekelle P:** Implementing PRISMA-P: recommendations for prospective authors. *Systematic Reviews* 2016 **5**:15

Section/topic	#	Checklist item	Information reported		Pages
			Yes	No	
**ADMINISTRATIVE INFORMATION**					
**Title**					
Identification	1a	Identify the report as a protocol of a systematic review	X		1
Update	1b	If the protocol is for an update of a previous systematic review, identify as such			n/a
**Registration**	2	If registered, provide the name of the registry (e.g., PROSPERO) and registration number in the Abstract	X		2
**Authors**					
Contact	3a	Provide name, institutional affiliation, and e-mail address of all protocol authors; provide physical mailing address of corresponding author	X		1
Contributions	3b	Describe contributions of protocol authors and identify the guarantor of the review	X		14
**Amendments**	4	If the protocol represents an amendment of a previously completed or published protocol, identify as such and list changes; otherwise, state plan for documenting important protocol amendments			n/a
**Support**					
Sources	5a	Indicate sources of financial or other support for the review	X		14
Sponsor	5b	Provide name for the review funder and/or sponsor	X		14
Role of sponsor/funder	5c	Describe roles of funder(s), sponsor(s), and/or institution(s), if any, in developing the protocol	X		14
**INTRODUCTION**					
**Rationale**	6	Describe the rationale for the review in the context of what is already known	X		4-5

For peer review only

BioMed Central
The Open Access Publisher

For peer review only - http://bmjopen.bmj.com/site/about/guidelines.xhtml

Section/topic	#	Checklist item	Information reported		Pages
			Yes	No	
**Objectives**	7	Provide an explicit statement of the question(s) the review will address with reference to participants, interventions, comparators, and outcomes (PICO)	X		5
		**METHODS**			
**Eligibility criteria**	8	Specify the study characteristics (e.g., PICO, study design, setting, time frame) and report characteristics (e.g., years considered, language, publication status) to be used as criteria for eligibility for the review	X		5
**Information sources**	9	Describe all intended information sources (e.g., electronic databases, contact with study authors, trial registers, or other grey literature sources) with planned dates of coverage	X		5
**Search strategy**	10	Present draft of search strategy to be used for at least one electronic database, including planned limits, such that it could be repeated	X		5-6
		STUDY RECORDS			
Data management	11a	Describe the mechanism(s) that will be used to manage records and data throughout the review	X		7
Selection process	11b	State the process that will be used for selecting studies (e.g., two independent reviewers) through each phase of the review (i.e., screening, eligibility, and inclusion in meta-analysis)	X		7
Data collection process	11c	Describe planned method of extracting data from reports (e.g., piloting forms, done independently, in duplicate), any processes for obtaining and confirming data from investigators	X		8
**Data items**	12	List and define all variables for which data will be sought (e.g., PICO items, funding sources), any pre-planned data assumptions and simplifications	X		8
**Outcomes and prioritization**	13	List and define all outcomes for which data will be sought, including prioritization of main and additional outcomes, with rationale	X		8
**Risk of bias in individual studies**	14	Describe anticipated methods for assessing risk of bias of individual studies, including whether this will be done at the outcome or study level, or both; state how this information will be used in data synthesis	X		8
		DATA			
**Synthesis**	15a	Describe criteria under which study data will be quantitatively synthesized	X		8
	15b	If data are appropriate for quantitative synthesis, describe planned summary measures, methods of handling data, and methods of combining data from studies, including any planned exploration of consistency (e.g., I^2, Kendall's tau)	X		8-9
	15c	Describe any proposed additional analyses (e.g., sensitivity or subgroup analyses, meta-regression)	X		8-9
	15d	If quantitative synthesis is not appropriate, describe the type of summary planned	X		8-9

BioMed Central
The Open Access Publisher

Section/topic	#	Checklist item	Information reported		Pages
			Yes	No	
**Meta-bias(es)**	16	Specify any planned assessment of meta-bias(es) (e.g., publication bias across studies, selective reporting within studies)	X		8
**Confidence in cumulative evidence**	17	Describe how the strength of the body of evidence will be assessed (e.g., GRADE)	X		8

For peer review only

BioMed Central
The Open Access Publisher
